# Outcomes and factors of elbow arthroscopy upon returning to sports for throwing athletes with osteoarthritis

**DOI:** 10.1186/s13018-018-0992-x

**Published:** 2018-11-07

**Authors:** Shun-Wun Jhan, Wen-Yi Chou, Kuan-Ting Wu, Ching-Jen Wang, Ya-Ju Yang, Jih-Yang Ko

**Affiliations:** 1 Department of Orthopedic Surgery, Kaohsiung Chang Gung Memorial Hospital and Chang Gung University College of Medicine, 123 Ta Pei Road, Niao Sung Dist, Kaohsiung, Taiwan; 2 Center for Shockwave Medicine and Tissue Engineering, Department of Medical Research, Kaohsiung Chang Gung Memorial Hospital and Chang Gung University College of Medicine, Kaohsiung, Taiwan

**Keywords:** Elbow arthroscope, Athletes, Returning to sports, Osteoarthritis

## Abstract

**Background:**

Elbow arthroscopy had good functional outcome for throwing athletes. Returning to sports is a major concern for all athletes, but only a few reports have investigated the clinical factors related to the duration of returning to sports. The present study evaluates the efficacy of elbow arthroscopic surgery on throwing elbows with osteoarthritis and defines the clinical factors related to the duration of the returning to sports.

**Methods:**

This was a retrospective study with fifteen active baseball throwing athletes with elbow osteoarthritis who were treated with elbow arthroscopy. Perioperative clinical factors were analyzed for functional outcomes. A multiple linear regression analysis was used to analyze the clinical factors associated with the duration of returning to training and sports.

**Results:**

The 15 patients’ mean age was 27 years. The mean follow-up time was 2.6 years. The mean procedural complexity was 3.1 ± 1.6 (range 1–6). The elbow total range of motion (ROM) improved significantly from 100.7 ± 28.7° to 125.7 ± 18.5° (*p* = 0.001). The terminal flexion range of the elbow increased significantly from 116.0 ± 22.6° to 130.0 ± 13.2° (*p* = 0.001), and the terminal extension range improved from 15.3 ± 11.1° to 4.3 ± 5.9° (*p* = 0.001). Before the operation, the average subjective patient outcome for return to sports (SPORTS) score was 3.4 ± 1.5, which increased significantly to 9.67 ± 0.45 (*p* = 0.003) at the last follow-up. The multiple linear regression analysis revealed that higher procedural complexity hinders the athletes from returning to competition.

**Conclusions:**

Elbow arthroscopy offered highly satisfactory results in the throwing elbows of elite athletes and significantly improved the range of motion and SPORTS score. The procedural complexity was significantly related to the duration of returning to competition. Early and aggressive arthroscopic intervention is recommended for elite throwing athletes with elbow osteoarthritis who fail to respond to conservative treatment.

## Background

Common causes of elbow arthritis include primary osteoarthritis, septic arthritis, post-traumatic arthritis, rheumatoid arthritis, crystalline arthropathy, and hemophilia [1]. Throwing athletes sustain consistent valgus extension overload stress on the elbow, which often leads to early traumatic arthritis due to the high demands in their daily activities. Chronic overuse with repetitive micro-trauma often results in subsequent scarring, contracture, and osteoarthritic changes [2, 3].

Valgus extension overload syndrome (VEOS) is a condition characterized by pathology in lateral radiocapitellum compression, medial collateral ligament tension, and posterior extension overload. Despite the increasing ulnar collateral ligament tears, osteoarthritis of the elbow is more common in throwing athletes [4, 5]. The clinical manifestation includes pain, catching or locking sensations, limited range of motion (ROM), and sensory paresthesia. The pathological changes within the elbow articulation include cartilage fragmentation, osteophyte formation, loose bodies within the joint, and capsular contracture.

The treatment options for osteoarthritis of the throwing elbow include anti-inflammatory medicine, activity modification with active rest, physical therapy, flexor-pronator strengthening, platelet-rich plasma injection, and arthroscopic debridement [6]. Due to the earlier recovery and its less invasive nature, elbow arthroscopic surgery has become a mainstay among surgical interventions when conservative treatments for the throwing elbow have failed. Compared to the shoulder arthroscopy in common throwing shoulder diseases, such as superior labral anterior-posterior lesion or rotator cuff tear, the result of elbow arthroscopy is superior in return to sports rate [7–9]. The indications for elbow arthroscopy include debridement for osteoarthritis, removal of loose bodies, synovectomy for inflammatory arthritis, contracture release, and osteochondral defect treatment [10, 11]. Complications related to elbow arthroscopy include superficial wound infection, wound complication, transient sensory paresthesia, deep intra-articular infection, persistent drainage, heterotrophic ossification, vascular injuries, and peripheral nerve injuries [12–14].

Previous reports have shown that elbow arthroscopy improves pain relief and the range of motion. It also has good functional outcomes and rates of returning to sports, which is a major concern for all athletes [15–17]. However, few reports have investigated the clinical factors related to the duration of returning to sports, which can be divided into returning to training and returning to competition. Therefore, the purpose of the present study is to evaluate the efficacy of elbow arthroscopic surgery in throwing elbows with osteoarthritis and to define the clinical factors related to the duration of returning to sports.

## Materials and methods

Since 2014, elbow arthroscopic debridement was used to treat athletes with elbow pain due to osteoarthritis who failed to respond to rest, oral medication, and physiotherapy for more than 3 months. The patients recruited were active overhead athletes who participated in a professional ball club or national team for at least 1 year. Osteoarthritis of the elbow was identified and classified radiologically using the Hasting and Rettig elbow osteoarthritis classification system. This system is a useful tool for predicting the surgical outcome of arthroscopic debridement for primary elbow osteoarthritis [18]. The system also shows substantial intraobserver and interobserver reliability for primary elbow osteoarthritis [19].

The diagnosis was initially made according to the clinical presentation and plain radiographs. Ultrasound or magnetic resonance imaging (MRI) was used to confirm the diagnosis and to exclude the possibility of ulnar collateral ligament tears or other conditions, including tears of the common extensor tendon or common flexor tendon. Contraindications for elbow arthroscopic included prior trauma, surgical scarring, and previous ulnar nerve transposition. All surgeries were performed by one orthopedic surgeon (W.Y.C.) who had subspecialty training in shoulder and elbow arthroscopy.

General demographic data were recorded, including age, gender, sport, affected elbow, and stage of elbow osteoarthritis. The preoperative factors recorded for the analysis were the duration of symptoms, preoperative terminal flexion, extension and ROM, and scores on the “subjective patient outcome for return to sports” (SPORTS) scale. The SPORTS score is a scoring system that is specifically designed to assess the return to sports, the level of performance, and the degree of residual impairment associated with doing sports [9]. It ranks the level of performance using five scales. Players receive a score of 10 if they can perform the same sports at the same level of effort and performance as before the onset of impairment and with no pain. Players who sustain mild pain receive a score of 9. Players who can perform the same sports at the same level of effort but reduced performance level compared to before onset of impairment receive a score of 6. Players who perform the same sports but at reduced levels of effort and performance compared to before the onset of impairment receive a score of 3. Players who are unable to return to the same sport receive a score of 0. A previous report shows that the SPORTS score is a valid and reliable scoring system for assessing the functional outcome and quantifying the return to sports [20].

The intraoperative factors examined were the olecranon process/fossa spurs, loose bodies, capitellum chondromalacia, and procedural complexity. The procedural complexity scale was adopted as one of the prognostic factors. This scale, which was first developed by Nelson et al. in 2014, ranges from 1 to 9, and its contributing factors include procedural specifics (scored as 1–5 points), tourniquet time (scored as 0–2 points), and the number of portals used (scored as 0–2 points). The procedural specifics included limited debridement, extensive debridement, capsular release, and osteocapsular arthroplasty, ranging from 1 point to 4 points, and release of posterior band of medial collateral ligament or medial epicondylectomy had an additional 1 point. The tourniquet time less than or equal to 60 min got 0 point and more than 90 min got 2 points. The portal number less than or equal to 2 got 0 point and more than 4 got 2 points [21]. Total complexity scores less than 4 are considered low, and scores greater than 5 are high. The postoperative factors include postoperative terminal flexion, extension and total ROM, and SPORTS scores.

All of these factors were utilized for the outcome assessment and to match the relationship of duration of returning to training and duration of returning to competition. The definition of duration of returning to training is the interval that athletes return to training without disruption by the symptoms after the surgery. Returning to competition was defined as the duration that athletes returned to the game according to the official game record after the surgery.

### Arthroscopic approach and rehabilitation

The patients were placed in a lateral decubitus position under general anesthesia. The operative arm was supported with an arm scaffold to allow complete arthroscopic examination of the elbow joint without antecubital fossa impingement. A tourniquet was applied to all patients to control bleeding. Standard 30° 4.5-mm arthroscope equipment was used.

**Fig. 1:**
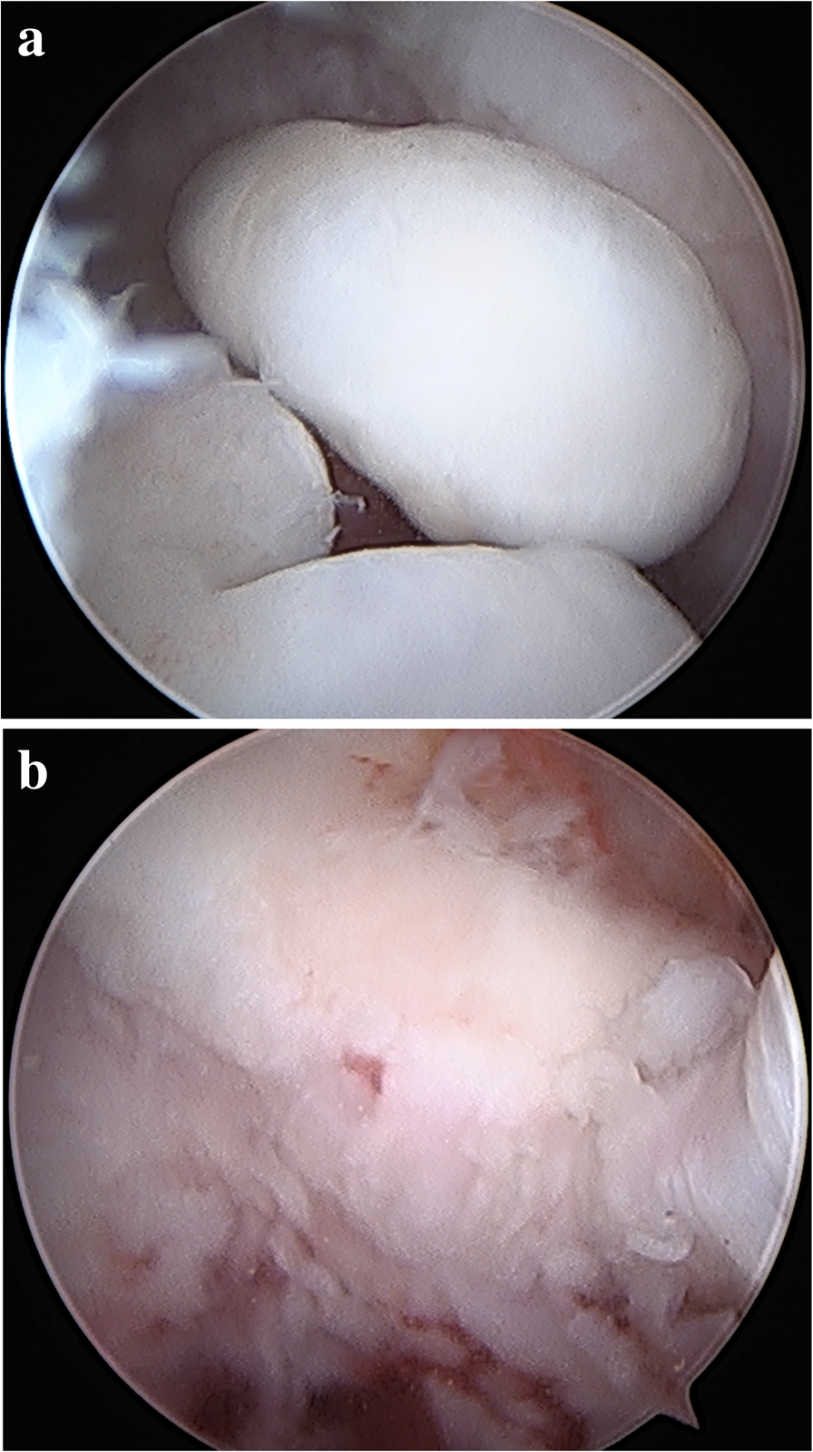
**a** Multiple loose bodies were found in the olecranon fossa. **b**: An olecranon fossa spur was identified after removal of loose bodies

Before the beginning of the operation, we mapped the course of the ulnar nerve and marked bony landmarks including the medial epicondyle, lateral epicondyle, radiocapitellar joint, and olecranon. Before portal placement, 20 ml of normal saline was injected to inflate the joint through an 18-gauge needle. Usually, a midlateral portal was created first to evaluate the radiocapitellum joint and olecranon fossa. An anterior lateral portal or posterior portal was created to facilitate the debridement, capsular lysis, removal of loose bodies, and the excision of osteophytes. The capsular release was also carried out based on the intraoperative findings and usually started from the posterior compartment to medial/lateral, sometimes anterior capsule if necessary. The presented cases revealed common intraoperative findings, including loose bodies and olecranon fossa spurs (Fig. 1a, b).

The patients were encouraged to perform passive or active-assisted motion on the next day after the surgery if the pain and swelling were tolerable. Aggressive active motions of the elbow were carried out in the third week post-operation, including pronation/supination and flexion/extension. Partial resistance training with an elastic rope or tubing started in the fourth to sixth weeks post-operation. The return to interval throwing usually started in the seventh week post-operation.

### Statistical analysis

All statistical analyses were performed using the SPSS software package (version 22.0; SPSS, Chicago, IL). A normality test of each variable was performed using the Shapiro-Wilk test, and comparisons of these variables were made with nonparametric tests (*P* < 0.05). The Wilcoxon signed-rank test was used to compare pre-operative and post-operative functional scores. Multiple linear regression analysis was used to determine the relative significance of each clinical factor associated with the duration of returning to training and sports.

## Results

From January 2014 to December 2016, we used elbow arthroscopic debridement and release to treat athletes with sustained elbow pain due to osteoarthritis who failed to respond to oral medication, physiotherapy, and other conservative treatments for more than 3 months. After the exclusion of two athletes who were lost to follow-up within 6 months, a total of 15 throwing elbows were recruited in the analysis. There were 12 professional baseball players and 3 amateurs with 12 right elbows and 3 left elbows involved. The mean age was 27 years (range 19–34 years). The mean follow-up time was 2.6 years (range 1.5–3.5 years) (Table 1).

**Table 1:** Demographic characteristics

Case	Age	Gender	Involved elbow	Sport	Level	Stages of osteoarthritis	Procedural complexity	Follow-up (years)
1	30	M	R	Baseball	Professional	II	1	3.5
2	33	M	L	Baseball	Professional	I	3	3.5
3	31	M	R	Baseball	Professional	I	4	3.1
4	19	M	L	Baseball	Semi-professional	I	3	3.1
5	24	M	R	Baseball	Professional	I	3	3
6	21	M	L	Baseball	Semi-professional	I	4	2.9
7	27	M	R	Baseball	Professional	I	5	2.8
8	27	M	R	Baseball	Professional	II	4	2.8
9	30	M	R	Baseball	Professional	I	5	2.7
10	28	M	R	Baseball	Professional	I	2	2.5
11	34	M	R	Baseball	Professional	I	2	2.4
12	27	M	R	Baseball	Professional	I	2	2.1
13	26	M	R	Baseball	Professional	II	6	1.5
14	20	M	R	Baseball	Semi-professional	I	1	1.5
15	26	M	R	Baseball	Professional	I	1	1.5

The mean procedural complexity was 3.1 ± 1.6 (range 1–6). Before the operation, the duration of symptoms that kept the athletes from participating in routine training or competition was 7.9 ± 3.1 months (range 4–12 months) (Table 2). Regarding the stages of elbow osteoarthritis, three patients sustained grade II osteoarthritis, and the rest of the 12 elbows had grade I osteoarthritis. The preoperative ROM was 100.7 ± 28.7° (range 45–140°).

**Table 2:** Functional outcome assessment

	Terminal flexion		Terminal extension		Total ROM		SPORTS		Duration of symptoms (months)	Duration of returning to training (months)	Duration of returning to sports (months)
Case	Pre-op	Post-op	Pre-op	Post-op	Pre-op	Post-op	Pre-op	Post-op			
1	125	135	0	0	125	135	3	10	6	0.5	4
2	90	135	10	0	80	135	3	10	12	1	5
3	125	135	10	0	115	135	3	10	6	2	6
4	125	135	10	0	115	135	6	10	5	2	3
5	70	130	25	10	45	120	3	10	8	1	2
6	130	135	40	10	90	125	3	9	12	4	6
7	90	130	25	10	65	120	0	10	7	3	6
8	100	100	30	15	70	85	6	9	12	0.25	6
9	135	140	10	0	125	140	3	10	6	5	6
10	140	140	10	5	130	135	3	9	6	3	4
11	140	140	20	0	120	140	6	10	12	2	3
12	130	135	10	0	120	135	3	10	12	3	5
13	90	100	20	15	70	85	3	9	5	0.75	6
14	140	140	0	0	140	140	3	10	4	2	3
15	110	120	10	0	100	120	3	10	6	0.25	3
Mean	116.0 ± 22.6° (70–140°)	130.0 ± 13.2° (100–140°)	15.3 ± 11.1° (0–40°)	4.3 ± 5.9° (0–15°)	100.7 ± 28.7° (45–140°)	125.7 ± 18.5° (85–140°)	3.4 ± 1.5 (0–6)	9.67 ± 0.45 (9–10)	7.9 ± 3.1 (4–12)	2.0 ± 1.5 (0.25–5)	4.5 ± 1.5 (2–6)
*P* value	0.001^a^		0.001^a^		0.001^a^		0.003^a^				

*ROM* range of motion, *SPORTS* subjective patient outcome for return to sports, *Pre-op* preoperative, *Post-op* postoperative

^a^A *p* value of < 0.05 was considered to be statistically significant

The intraoperative findings showed that 14 out of 15 patients (93.3%) had olecranon process spurs, and 5 patients (33.3%) had olecranon fossa impingement spurs. There were 10 patients (66.7%) who had loose bodies in the olecranon fossa and 4 patients (26.7%) with capitellum chondromalacia. The elbow terminal flexion range significantly increased from 116.0 ± 22.6° to 130.0 ± 13.2° (*p* = 0.001), and the terminal extension range also improved from 15.3 ± 11.1° to 4.3 ± 5.9° (*p* = 0.001). The total elbow ROM improved significantly from a mean of 100.7 ± 28.7° to 125.7 ± 18.5° (*p* = 0.001).

Before the operation, the average SPORTS score was 3.4 ± 1.5, which increased significantly to 9.67 ± 0.45 (*p* = 0.003) at the last follow up. In this analysis, all patients had SPORTS score less than 6 before the operation and had returned to the same level of competition after elbow arthroscopy as of the last follow-up. The mean follow-up interval was 2.59 years (range 1.5–3.5 years). The mean interval of returning to training was 2.0 ± 1.5 months (range 0.25–5 months) postoperatively, and the mean interval of returning to competition was 4.5 ± 1.5 months (range 2–6 months). There were no perioperative complications in this series, and no further surgical intervention was required at the last follow-up (Table 2).

In the multiple linear regression analysis, all the preoperative, intraoperative, and postoperative factors did not show significance regarding the duration of returning to training (Table 3). However, the procedural complexity demonstrated an influence on returning to competition (Table 4). The results implied that the complexity of elbow osteoarthritis is significantly related to the duration of returning to competition.

**Table 3:** Multiple linear regression analysis of factors associated with duration of return to training

Variable	Coefficients	S.E.	*p* value
Preoperative factors			
Duration of symptoms	0.050	0.179	0.808
Preoperative terminal Flexion	0.016	0.039	0.729
Preoperative terminal extension	0.037	0.064	0.621
Preoperative ROM	0.017	0.013	0.204
Preoperative SPORTS	0.009	0.348	0.981
Intraoperative factors			
Procedural complexity	0.324	0.468	0.560
Olecranon fossa spur	0.947	1.258	0.530
Olecranon process spur	2.150	2.740	0.515
Loose bodies	− 0.497	1.141	0.706
Capitellum chondromalacia	1.686	1.846	0.457
Postoperative factors			
Postoperative terminal flexion	0.092	0.065	0.293
Postoperative terminal extension	− 0.080	0.222	0.753
Postoperative ROM	0.037	0.019	0.074
Postoperative SPORTS	− 1.248	2.108	0.614

*S.E* standard error of coefficient, *Pre-op* pre-operative, *Post-op* post-operative, *ROM* range of motion

*SPORTS* subjective patient outcome for return to sports

**Table 4:** Multiple linear regression analysis of factors associated with duration of return to competition

Variable	Coefficients	S.E	*p* value
Preoperative factors			
Duration of symptoms	0.149	0.071	0.171
Preoperative terminal flexion	0.014	0.016	0.463
Preoperative terminal extension	− 0.024	0.026	0.452
Preoperative ROM	− 0.009	0.014	0.542
Preoperative SPORTS	− 0.355	0.139	0.125
Intraoperative factors			
Procedural complexity	1.406	0.186	0.017^a^
Olecranon fossa spur	1.198	0.502	0.140
Olecranon process spur	− 3.590	1.092	0.081
Loose bodies	0.104	0.455	0.840
Capitellum chondromalacia	− 1.446	0.736	0.188
Postoperative factors			
Postoperative terminal flexion	− 0.017	0.026	0.581
Postoperative terminal extension	− 0.164	0.088	0.205
Postoperative ROM	− 0.029	0.021	0.195
Postoperative SPORTS	− 1.567	0.841	0.203

*S.E.* standard error of coefficient, *Pre-op* pre-operative, *Post-op* post-operative, *ROM* range of motion

*SPORTS:* subjective patient outcome for return to sports

^a^A *p* value of < 0.05 was considered to be statistically significant

## Discussion

In this series, we found consistent satisfactory results of arthroscopic debridement and release regarding osteoarthritis of the throwing elbow, as in previous reports. All the athletes could return to sports without complications in the mean follow-up period of 2.6 years. The mean durations of returning to training and competition were 2.0 ± 1.5 and 4.5 ± 1.5 months, respectively, which could be a reference for athletes and coaches to estimate the duration of returning to play (Table 2). Another principle finding is that the procedural complexity was significantly related to the duration of returning to competition, which indicated that the complexity of elbow osteoarthritis hindered the interval of returning to competition. Early and aggressive intervention for throwing elbows with osteoarthritis should be considered in patients who fail to respond to conservative treatments for more than 3 months.

According to Morrey et al., the elbow motion necessary for most daily activities in a functional arc of motion, which ranges from 30 to 130° [22]. Conservative treatments such as medication, splinting, and rehabilitation should be considered in patients with early osteoarthritis of the elbow or minor elbow contracture. However, the functional arc of motion might not be applicable to patients with highly demanding circumstances, such as throwing athletes. In this subpopulation, arthroscopic debridement and capsular release offer a minimally invasive modality to relieve symptoms and allow an earlier return to sports compared to traditional open procedures.

Tucker et al. concluded that elbow arthroscopy performed by an experienced doctor can produce better results than open release [17]. Previous studies show that elbow arthroscopic debridement and arthrolysis contribute significantly to the improvement of the elbow’s ROM. Somanchi et al. reported improvements in elbow flexion and extension of 6° and 12.5° after elbow arthroscopic lysis, respectively [23]. Nguyen et al. retrospectively reviewed 22 patients who underwent elbow arthroscopy with an improvement of 19° terminal flexion and 19° terminal extension after 1 year of follow-up [16]. Meluzinová et al. and Cefo et al. both reported significant improvements in the average elbow ROM after arthroscopic treatment for the post-traumatic stiff elbow joint [24, 25]. Adams et al. reviewed 41 patients with 42 cases of primary osteoarthritis of the elbow who received arthroscopy for more than 2 years. The mean flexion, extension, and supination had significant improvements of 14.3°, 13.0°, and 7.9°, respectively [26]. In the present analysis, the improvement of ROM, terminal flexion, and extension correspond to the previous reports, but the follow-up was longer (mean 2.6 years).

Risks of elbow arthroscopy are believed to be related to the complications of the procedure. Kelly et al. reported that the minor complication rate after arthroscopic procedures was about 11%, and the complications would resolve spontaneously. Major complications such as deep joint infection were rare (0.8%) [12]. Nelson et al. reported that the complication rate of elbow arthroscopy was about 14% of cases. The major complication rate was about 5%, and repeated surgeries were needed in these cases [21]. Some investigators showed one superficial portal site infection in 14 patients after a 1-year follow-up [15]. Blonna et al. reported three cases of delayed-onset ulnar neuropathy in 26 patients who received arthroscopic treatment for terminal extension restoration. Two of them required further ulnar nerve transposition surgery [9]. It is reported that the elbow arthroscopy is limited in treating posterolateral rotatory instability and septic arthritis [11]. In addition, ulnar nerve compromise also should be highlighted for the athletes with the history of ulnar nerve transposition. In the present series, all of the elbows were sports-related osteoarthritis in which the results of arthroscopy are encouraging and our results were in line with the previous reports. Although the anterior bundle of ulnar collateral ligament contributes the majority of medial elbow stability, Terzini et al. also reported that the posterior bundle provides the stability in higher flexion angle [27]. The osteoarthritic elbow with collateral ligament injury-related instability was excluded from the present study since the ligamentous reconstruction, such as ulnar collateral ligament reconstruction, were done with extra-articular open procedure nowadays. There were no complications in the present study, which may due to the limited number of cases, the exclusion of elderly patients and patients with post-traumatic osteoarthritis, and the relatively low procedural complexity.

Ward et al. retrospectively reviewed 36 athletes who received elbow arthroscopy and reported that the most commonly treated lesions were loose bodies and impingement spurs, which were compatible with the present analysis. There was also a significant improvement in the subjective functional outcome score [28]. Somanchi reported an 87% satisfaction rate in 26 elbow arthroscopic patients in a 2-year follow-up study [23]. Blonna et al. revealed that 25 out of 26 athletes were restored to normal or near-normal function. Most importantly, 90% of patients returned to the same performance level as before the onset of impairment [9]. Even in advanced elbow capitellar osteochondritis dissecans, surgical intervention with autologous osteochondral mosaicplasty also had a high return to pre-injury competitive level rate (91%) [29]. In our study, all 15 athletes could return to the same competition level after the operation without recurrence in the mean follow-up of 2.6 years. The mean postoperative SPORTS score was 9.67, which is significantly improved from the score of 3.4, which indicated a high subjective patient outcome regarding the return to sports.

The multiple linear regression analysis excluded postulated clinical factors that might hinder the durations of returning to training and competition, including the duration of symptoms, terminal flexion and extension, total ROM, olecranon spurs, loose bodies, capitellum chondromalacia, and SPORTS score. But the procedural complexity scale was significantly related to the return to competition. This points out the complexities of elbow osteoarthritis, such as the involvement of more than two compartments or extensive capsular release that requires longer surgical time. These are factors that hindered the athletes from returning to competition due to the highly intensive demands during games rather than training. In terms of procedure complexity scale, early arthroscopic intervention for the athletes with osteoarthritis of the elbow is recommended for the arthritis retardation and the early return to competition.

The present study had some limitations. First, the retrospective analysis had selection bias. Second, there was no control group, such as athletes without surgical intervention, which restricted the performance of a comprehensive comparison. Third, the limited case numbers weakened the statistical analysis. Fourth, the procedural complexity scale had subjective components. For example,the tourniquet time and portal number might be influenced by the operation room equipment condition and the surgeon’s experience since elbow arthroscopy is an operator-dependent procedure. However, there was a range between each point, which might reduce the subjective effect. In spite of the limitations, however, the present study offers the consistent results of arthroscopic treatment for throwing elbows. We also determined that the procedural complexity is a factor that hinders the return to competition.

## Conclusions

In conclusion, elbow arthroscopy offered consistent and highly satisfactory results in throwing elbows with osteoarthritis, as shown in the improvement of ROM, SPORTS scores, and the 100% return to sports in a mean follow-up period of 2.6 years. The mean durations of returning to training and competition were 2 and 4.5 months, respectively, which could be a reference for practitioners to estimate the duration of returning to play. The procedural complexity was significantly related to the duration of returning to competition. Early and aggressive intervention for throwing elbows with osteoarthritis should be considered in athletes who have failed to respond to conservative treatments for more than 3 months.

## Abbreviations

MRI: Magnetic resonance imaging
ROM: Range of motion
SPORTS: Subjective patient outcome for return to sports
VEOS: Valgus extension overload syndrome
